# Association of Sensory Nerve Action Potential Amplitude and Velocity With Type 2 Diabetic Peripheral Neuropathy

**DOI:** 10.7759/cureus.46501

**Published:** 2023-10-04

**Authors:** Anwar H Siddiqui, Nazia Tauheed, Hamid Ashraf, Jamal Ahmad

**Affiliations:** 1 Physiology, Jawaharlal Nehru Medical College, Aligarh Muslim University, Aligarh, IND; 2 Anaesthesiology, Jawaharlal Nehru Medical College, Aligarh Muslim University, Aligarh, IND; 3 Endocrinology and Diabetes, Rajiv Gandhi Centre for Diabetes and Endocrinology, Aligarh Muslim University, Aligarh, IND

**Keywords:** early diagnosis, amplitude of action potential, nerve conduction study, motor nerve, sensory nerve, diabetic peripheral neuropathy

## Abstract

Background: There is ongoing controversy regarding the predominant type of nerve injury in diabetic peripheral neuropathy, whether it is demyelination or axonal degeneration.

Objective: This study aimed to investigate the association between nerve conduction study parameters, specifically nerve conduction velocity and the amplitude of the action potential, with diabetic peripheral neuropathy and determine their potential as early indicators of the condition.

Methods: A cross-sectional study was conducted involving diagnosed type 2 diabetes mellitus patients, who were divided into two groups: Group I (n = 111) with symptomatic diabetic peripheral neuropathy and Group II (n = 109) without clinically detectable peripheral neuropathy. Age and sex-matched healthy controls (n = 100) were also included. Nerve conduction velocity measurements were performed on both upper and lower limbs, with motor nerve conduction study focusing on the dominant side using the median and posterior tibial nerves and sensory nerve conduction study using the median and sural nerves.

Results: The nerve conduction studies revealed significantly lower sensory nerve action potential amplitudes and compound muscle action potential amplitudes in the median, posterior tibial, and sural nerves of the diabetic groups compared to the control subjects. Furthermore, these changes were more prominent in patients with peripheral neuropathy. Among the 220 diabetic patients analyzed, 135 (61.36%) exhibited nerve conduction abnormalities. The highest rate of abnormality was observed in the sural nerve, followed by the posterior tibial and median nerves. The most common abnormality detected in diabetic patients was a decrease in sensory nerve action potential, followed by a decrease in sensory nerve conduction velocity.

Conclusion: The study findings suggest an association between reduced sensory nerve action potential amplitude and diabetic peripheral neuropathy. These results highlight the potential of sensory nerve action potential and velocity as a sensitive indicator of peripheral neuropathy in diabetic patients.

## Introduction

Diabetes Mellitus is a major public health problem across the globe. It is one of the major non-communicable diseases having a huge impact on the physical and emotional quality of life. Among the multitude of complications of diabetes, diabetic peripheral neuropathy (DPN) stands out to be the most common complication. The prevalence of DPN in diabetic patients ranges from around 10.5% to 32.2% in the Indian population and as per Western studies, up to 50% of patients will eventually develop neuropathy during the course of their disease [1]. Diabetic peripheral neuropathy leads to sensory loss and damage to the limbs and is the leading cause of lower extremity amputations not related to an injury. Early diagnosis and management of DPN are extremely important as once frank symptoms appear, there are few effective therapeutic strategies [2]. Diabetic peripheral neuropathy is associated with various abnormalities, such as nerve fiber axonal degeneration, primary demyelination due to Schwann cell dysfunction, and secondary segmental demyelination. The distinction between demyelinating and axonal neuropathy can be established through nerve conduction studies (NCS) [3].

Nerve conduction studies comprise two crucial components: nerve conduction velocity and nerve conduction amplitude, which help distinguish between demyelinating neuropathy and axonal degeneration [4]. Demyelinating neuropathy is identified when the conduction velocity slows down by more than 40% of the normal mean, whereas the degree of reduction in amplitudes of sensory or motor compound action potential (SNAP or CMAP) to distal stimulation provides an indication of axonal degeneration [5]. Consequently, nerve conduction velocity predominantly reflects alterations in myelin, whereas the action potential amplitude serves as an indicator of axonal changes and the condition of the nerve fibers. The reduction in action potential amplitude suggests axonal damage and provides an estimate of the number of neural fibers activated by electrical stimulation [6].

A literature search reveals that most of the nerve conduction studies done on diabetic peripheral neuropathy patients mainly emphasize the reduction in nerve conduction velocity without giving any due importance to nerve conduction amplitude. Kimura et al. in 1979 reported increased latency and decreased conduction velocity of peroneal and tibial nerves in diabetics as compared to normal subjects [7]. Hoffman et al. in 2009 reported the conduction velocity of the peroneal nerve to be significantly slower in the diabetic subjects than in the control [8]. In 2014, Gauhar et al. conducted a study comparing nerve conduction velocities of motor and sensory nerves in patients with clinically detectable neuropathy and patients without neuropathy within the type 2 diabetes mellitus population. Their findings revealed a significant reduction in nerve conduction velocities in the lower limbs, while the upper limb nerve conduction velocities remained normal [9]. However, there are limited studies that have investigated and compared the amplitudes of sensory or motor compound action potential (SNAP or CMAP) in DPN [10,11].

Multiple sources in the literature suggest that nerve conduction velocity is a more variable parameter compared to action potential amplitude and is highly influenced by the techniques of measurement and associated comorbidities [12]. Conduction velocities are generally within the normal range or only mildly slowed in distal symmetrical polyneuropathy, the most common form of DPN. It has been reported that if conduction velocities are significantly less, the patient may have other causes of peripheral nerve demyelination superimposed on the more typical axonal loss seen in DPN [13]. Existing studies have mainly emphasized the assessment of nerve conduction velocity, while the amplitude of the action potential has not received significant attention in the context of diabetic peripheral neuropathy (DPN). This creates a notable research gap, as the nerve conduction amplitude offers valuable insights into the number of neural fibers activated by electrical stimulation and indicates the extent of axonal damage. By addressing this research gap and investigating the significance of nerve conduction amplitude in DPN, our study aims to contribute to the broader understanding of DPN pathophysiology and potentially enhance diagnostic approaches. Ultimately, this research has the potential to improve patient outcomes by enabling early identification and targeted management strategies for individuals at risk of developing DPN complications

In consideration of the above-mentioned content, the present study aimed to investigate which nerve conduction study parameter is better associated with diabetic peripheral neuropathy, the nerve conduction velocity or the amplitude of the action potential.

## Materials and methods

Setting and design

This institutional cross-sectional study was conducted in the Department of Physiology, Jawaharlal Nehru Medical College, in collaboration with the Rajiv Gandhi Centre for Diabetes and Endocrinology, Aligarh Muslim University, from April 2022 to October 2022. The study population included type 2 diabetic patients visiting the diabetes clinic outpatient department (OPD) of our institution. Patients were divided into two groups based on the clinical examination and neuropathy disability score: Group I included patients without diabetic peripheral neuropathy, while Group II included patients with clinically detectable diabetic peripheral neuropathy. Additionally, one hundred apparently healthy subjects were recruited as controls.

Sample size calculation and subject selection

The sample size was calculated using the following formula: (N) = ((Z1-a/2)2 ×P×Q)/ D2

Where, (Z1-a/2) =Standard normal variation (at 2SD of variation and at 95% confidence level), Z1-a/2=1.96; P=Prevalence of peripheral neuropathy in T2DM 16.6% [14]; Q=100-P; and D= Absolute error or precision (taken as 7%).

The calculated sample size (N) was 108 for each group, providing a power of 80% at a 95% confidence interval. However, initially, 125 type 2 diabetic patients were included in each group. After excluding patients with an inconclusive history of any coexisting cardiovascular disease, the final group sizes were as follows: Group I (n = 111), Group II (n = 109), and Group III (n = 100, healthy controls). Patient selection adhered strictly to the specified inclusion and exclusion criteria.

Inclusion and exclusion criteria

This cross-sectional study focused on Type 2 Diabetes Mellitus patients aged 30 to 60 years, as older patients may have an absent sural response and could confound the results. The diagnosis of diabetes in this study was determined based on the revised American Diabetes Association Criteria. This criterion includes specific thresholds for fasting plasma glucose and 2-hour postprandial plasma glucose during an oral glucose tolerance test, as well as the level of glycosylated hemoglobin (HbA1c) [15].

Patients who participated in the study provided informed consent for the nerve conduction study. However, patients with peripheral neuropathy of inflammatory and infectious etiologies (such as Carpal tunnel syndrome, Guillain-Barre syndrome, vasculitic neuropathies, and Hansen neuropathy) were excluded. Additionally, patients with neuropathies associated with nutritional deficiency (vitamin B12 deficiency), paraneoplastic syndromes, exogenous toxins, metals, and drugs were also excluded from the study.

Furthermore, patients with peripheral artery disease or evidence of limb ischemia were excluded to ensure the integrity of the nerve conduction study recordings. Patients with any form of trauma, skin lesions, or swelling in the course of nerves that could interfere with the nerve conduction study were not included in the study.

Ethical approval and clinical data collection

The study received ethical approval from the Institutional Ethical Committee, ensuring that the research adhered to ethical guidelines and protected the rights and well-being of the participants. Informed consent was obtained from all participants, indicating that they were fully aware of the study's purpose, procedures, potential risks, and benefits before agreeing to participate.

Patient demographic profiles, including gender, age, duration of diabetes, height, and weight, were collected through a structured history and examination using a pre-designed proforma. Additionally, blood pressure was measured three times using a standard mercury sphygmomanometer, and the measurements were averaged to obtain an accurate representation of the participant's blood pressure levels.

Patients experiencing symptoms such as paresthesia, burning, numbness, tingling, cramping, and aching were clinically evaluated for peripheral neuropathy using two assessment tools: the Diabetic Neuropathy Symptom Score (DNS) and the Neuropathy Disability Score (NDS). DNS has a maximum score of four points, whereas NDS has a maximum score of 10 points. A score of one or more in DNS and a score of six or more in NDS indicates the presence of neurological abnormalities [16,17].

Nerve conduction studies (NCS)

The nerve conduction study was conducted using Neuroperfect software on a computerized EMG/NCV/EP (electromyography, nerve conduction velocity, electrophysiology) system provided by Medicaid System, Chandigarh, India. The study followed standard surface stimulating and recording techniques, and NCV (nerve conduction velocity) measurements were taken in both upper and lower limbs on both sides of the body. For the motor nerve conduction study, the Median and Posterior tibial nerves of the dominant side were chosen, while the sensory nerve conduction study involved the Median and Sural nerves of the dominant side. However, the room was air-conditioned to prevent excessive ambient temperature. It is essential to note that patients were not subjected to excessively cold temperatures caused by air conditioning since prolonged exposure to colder environments, even in a tropical region like India, could potentially impact nerve conduction velocities, particularly sensory response. Misra and Kalita derived normal nerve conduction parameters in the north Indian population, which were used as references for comparison [18].

Biochemical analysis

Participants were instructed to visit the endocrinology laboratory after fasting for 10-12 hours overnight. Blood samples were collected to analyze fasting blood glucose, postprandial blood glucose, and glycosylated hemoglobin (HbA1c). Plasma glucose was measured using the glucose oxidase peroxidase enzymatic method, while HbA1c was estimated using the cation exchange resin method.

Statistical analysis

The data were analyzed using Statistical Package for the Social Science software, version 25.0 for Windows (IBM Corp., Armonk, NY). The normalcy of the data was assessed by Kolmogorov-Smirnov tests. Continuous variables were presented as mean ± standard deviation. A one-way analysis of variance (ANOVA) was utilized to compare continuous data among the different groups. Post hoc multiple comparisons were conducted using the Tukey-Kramer test to identify significant differences between specific groups. All statistical tests were two-tailed, and 95% confidence intervals were calculated. A p-value of less than 0.05 was considered statistically significant, indicating that there is a low probability of obtaining the observed results by chance alone.

## Results

Baseline clinical characteristics and laboratory data of the patients and the control included in the current analyses are shown in Table 1. There was no significant difference in the age, weight, and sex distribution among the three groups. Group I diabetic patients had significantly elevated levels of HbA1c, post prandial glucose, and total cholesterol as compared to the group II patients as well as the control. Group I patients had a significantly longer duration of diabetes as compared to Group II.

**Table 1:** Demographic and biochemical characteristics of all the study participants Data are means ± SD; P values shown in parenthesis * P < 0.05 vs control; #P < 0.05 vs. DM without DPN (one way ANOVA); DM: Diabetes Mellitus; DPN: Diabetic peripheral neuropathy

	Group I: Type 2 diabetic patients with peripheral neuropathy	Group II: Type 2 diabetic patients without peripheral neuropathy.	Group III: Healthy control
Number	111	109	100
Male: Female	59:52	61:48	58:42
Age (yrs)	48.6 ± 9.22	45.6 ± 8.42	38.8 ± 10.45
Weight (kg)	66.70 ± 8.88	62.78 ± 7.83	56.54 ± 6.92
Duration of Diabetes	10.5 ± 4.7 (#0.003)	4.57 ± 2.3	
HbA1C (%)	8.34 ± 2.24(*0.023, #0.004)	6.27 ± 2.11	5.18 ± 1.64
Fasting plasma glucose (mg/dL)	152.14 ± 20.79 (*0.001)	141.78 ± 24.77(*0.002)	87.35 ± 17.07
Post prandial glucose (mg/dL)	261.36 ± 28.22 (*0.003, #0.014)	191.76 ± 18.70 (*0.001)	124.50 ±15.06
Systolic blood pressure (mmHg)	138 ± 23.14	135.56 ± 22.67	119.75 ± 11.50
Diastolic blood pressure (mmHg)	84,2 ± 7.04	83.30 ± 11.82	79.85 ± 8.63
Total cholesterol (mg/dl)	218.36 ± 45.31 (*0.002, #0.012)	198.70 ± 31.55	172.64 ± 27.26
Triglycerides (mg/dl)	124.24 ± 46.12 (*0.021)	118.36 ± 37.84	105.82 ± 22.65

**Figure 1:**
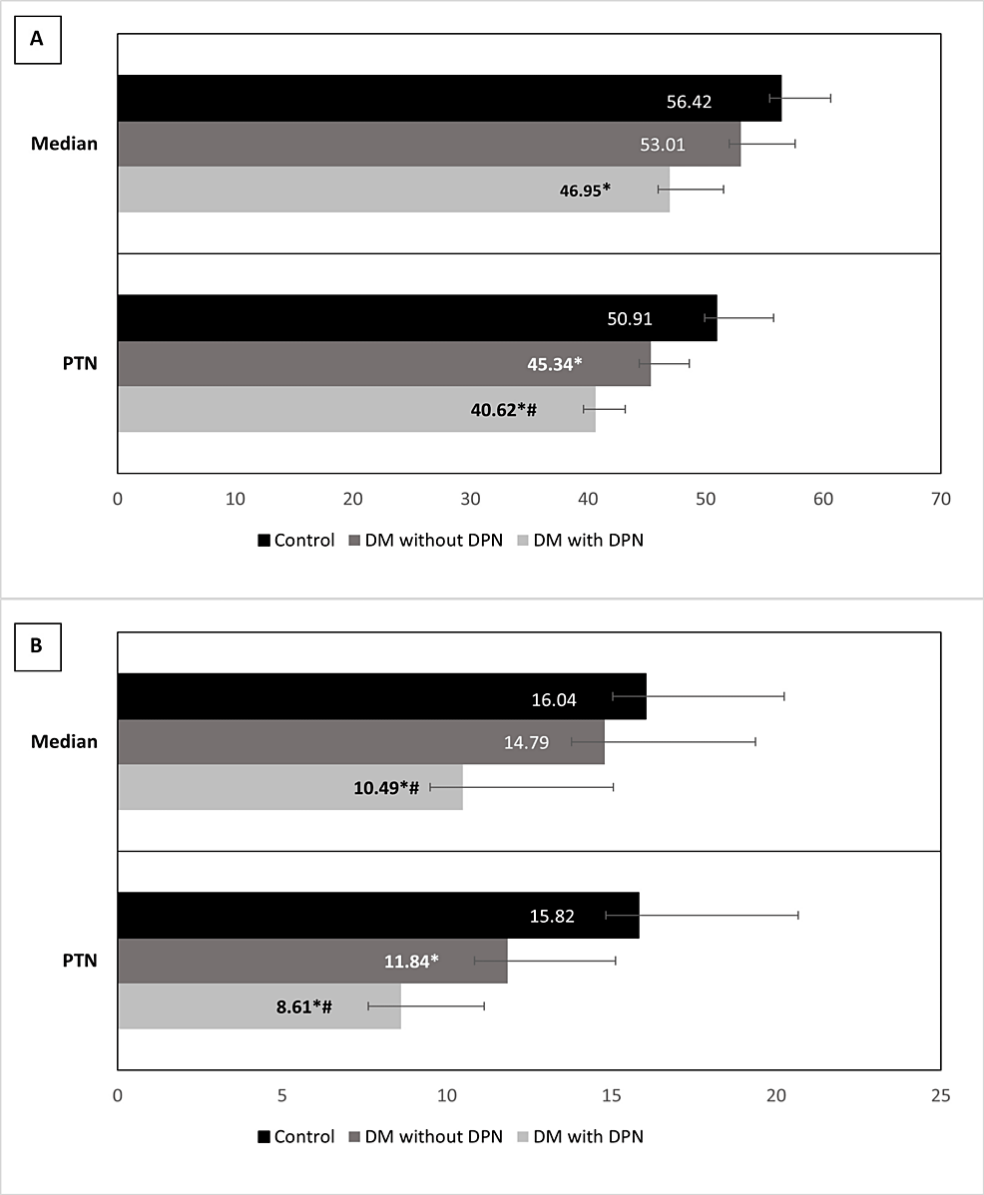
A comparison of motor nerve conduction velocity parameters in the three different groups Panel A shows the motor nerve conduction velocity (MNCV), while Panel B depicts the compound muscle action potential (CMAP). *P < 0.05 vs control; #P < 0.05 vs. DM without DPN (one way ANOVA); DM: Diabetes Mellitus; DPN: Diabetic peripheral neuropathy

Motor nerve conduction analysis was done in the dominant median and the posterior tibial nerve. Comparative analysis using one-way ANOVA revealed that the motor nerve conduction velocity of the median nerve was significantly reduced in the group I patients as compared to healthy controls, however, there wasn’t any significant difference with respect to group II patients. Motor nerve conduction velocity of the posterior tibial nerve was significantly reduced in the diabetic patients, both with and without peripheral neuropathy, as compared to healthy controls, with group I patients also showing a significantly reduced velocity compared to group II (Figure 1A). The compound muscle action potential (CMAP) of the median nerve in the diabetic patients with peripheral neuropathy (group I) was significantly decreased as compared to the patients without peripheral neuropathy (group II) and healthy controls. No significant difference was observed between group II and healthy controls. The CMAP of posterior tibial nerves of group 1 patients was, however, significantly decreased as compared to both group II and healthy controls (Figure 1B).

**Figure 2:**
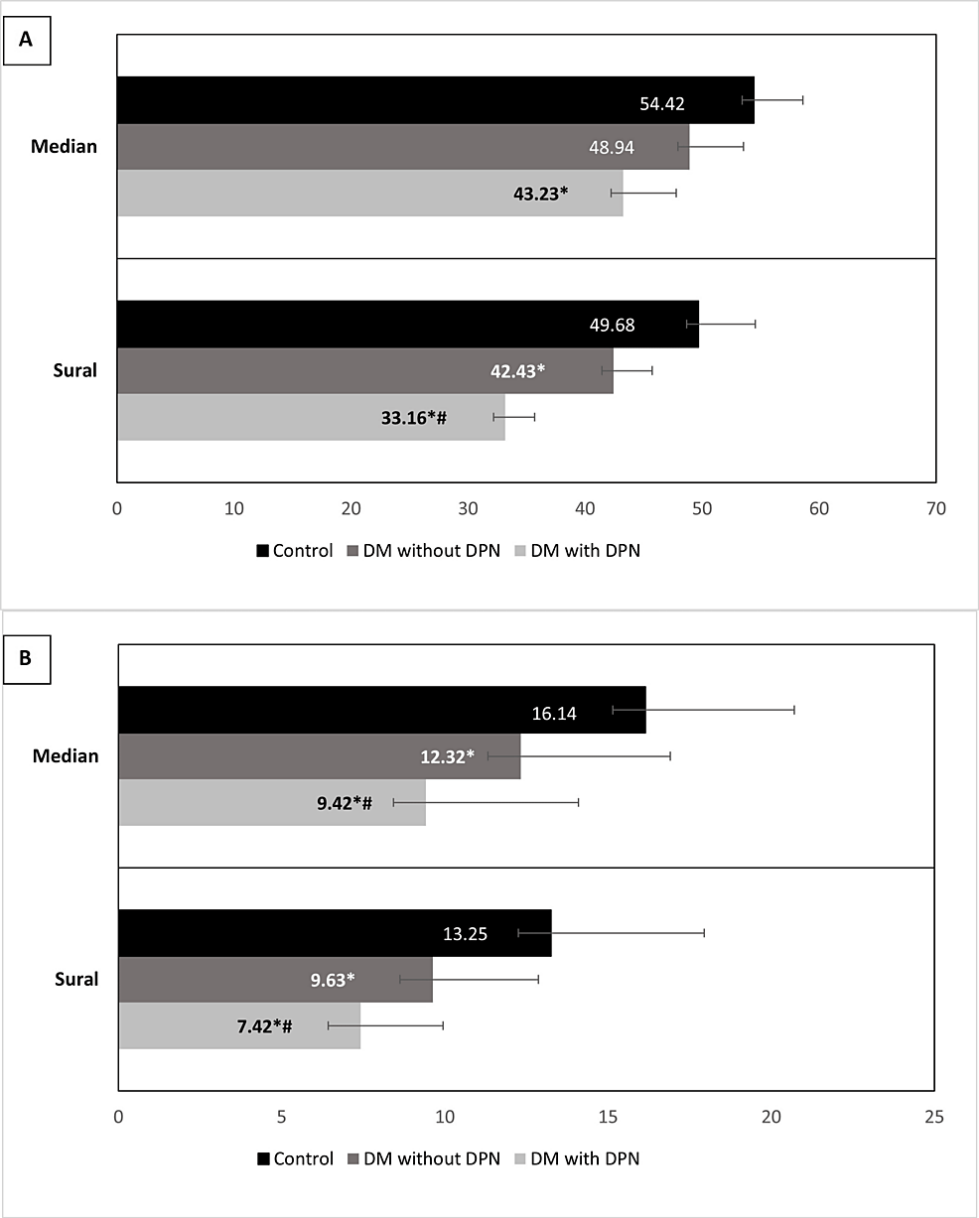
Comparison of sensory nerve conduction velocity parameters in the three different groups Panel A shows the sensory nerve conduction velocity (SNCV), while Panel B depicts the sensory nerve action potential amplitude (SNAP). *P < 0.05 vs control; #P < 0.05 vs. DM without DPN (one way ANOVA); DM: Diabetes Mellitus; DPN: Diabetic peripheral neuropathy

Sensory nerve conduction analysis revealed a significant decrease in the median nerve conduction velocity of the group I patients compared to the healthy controls. No significant difference was observed when compared with group II. However, the sural nerve sensory conduction velocity of the group I patients significantly decreased as compared to both group II and controls (Figure 2A). The sensory nerve action potential (SNAP) analysis showed a significantly decreased SNAP of the median as well as the sural nerve in the group I patients as compared to the other groups (Figure 2B).

Out of the 220 diabetic patients (comprising both Group I and Group II), 135 individuals (61.36%) exhibited nerve conduction abnormalities. Among the nerves assessed, the sural nerve showed the highest rate of abnormality, followed by the posterior tibial nerve and the median nerve. The most common nerve conduction abnormality detected in diabetic patients was related to the sensory nerve action potential (SNAP), followed by sensory nerve conduction velocity (SNCV), while the compound motor action potential (CMAP) showed the least frequency of abnormalities. Particularly, the sural nerve sensory nerve action potential demonstrated the highest rate of abnormalities among the assessed nerve conduction parameters (Table 2).

**Table 2:** Percentage of abnormality detected in both motor and sensory nerve conduction studies among diabetic patients with and without peripheral neuropathy (n=220). A Chi-square test was used to compare the two samples. MNCV: Motor nerve conduction velocity; CMAP: Compound muscle action potential; SNCV: Sensory nerve conduction velocity; SNAP: Sensory nerve action potential

Nerves	Percentage of abnormality detected	Motor nerve conduction abnormality (%)		Sensory nerve conduction abnormality (%)			
		MNCV	CMAP	SNCV	SNAP	χ2	P
Median	53.63	24.4	32.5	42.9	56.8	386.04	<0.05
Posterior Tibial Nerve	57.27	21.5	37.4	34.6	48.4	292.73	<0.05
Sural	61.81	23.6	43.34	44.2	58.4	327.36	<0.05

## Discussion

Diabetic peripheral neuropathy (DPN) is a complex disorder characterized by the impairment of different types of nerve fibers in individuals with diabetes. Early diagnosis of DPN is challenging due to its insidious onset and lack of symptoms in the initial stages. Electrophysiological studies, particularly nerve conduction studies, have emerged as valuable tools in detecting subclinical pathological changes in early DPN. These studies assess the ability of peripheral nerves to conduct electrical signals and identify abnormalities in the myelin sheath, nodes of Ranvier, and axons [19].

In our study, we recruited 250 patients with type 2 diabetes, categorized into two groups: Group I with symptomatic DPN and Group II without clinically detectable peripheral neuropathy. After excluding patients with incomplete data, the final analysis included 220 participants (111 with neuropathy and 109 without neuropathy). The analysis revealed that 61.36% of all diabetic patients exhibited nerve conduction abnormalities. Our findings are consistent with a study by Zhang et al., 2014, which reported a total abnormal nerve conduction rate of 71.6% in diabetic groups [20].

Among the diabetic patients with peripheral neuropathy, we observed significantly reduced motor and sensory nerve conduction velocities, as well as decreased amplitude of compound muscle action potentials (CMAP) and sensory nerve action potentials (SNAP) in all studied nerves, compared to healthy controls. Furthermore, the changes were more pronounced in patients with peripheral neuropathy, indicating the exacerbation of lesions in sensory fibers of the median and sural nerves, as well as the axons and myelin sheath of motor fibers of the posterior tibial nerves.

Interestingly, even in diabetic patients without clinically detectable peripheral neuropathy, we found significantly reduced sensory and motor conduction velocities and decreased amplitude of sensory and motor action potentials in the posterior tibial and median nerves compared to controls. The findings indicate that even in asymptomatic diabetic patients, there is evidence of impaired sensory and motor axons and myelin sheath at the distal end. This suggests that diabetic peripheral neuropathy may be present subclinically, even before the onset of noticeable symptoms. Therefore, nerve conduction studies can be considered an effective tool for the early detection of diabetic peripheral neuropathy, enabling prompt management and prevention of long-term complications such as limb amputation.

Our study also sheds light on the pattern and type of nerves commonly affected in diabetic peripheral neuropathy. The study observed that sensory nerves were more susceptible to damage than motor nerves in both diabetic patient groups. Specifically, the sural nerve and posterior tibial nerves in the lower limbs were more affected than the median nerve in the upper limb. This suggests that diabetic peripheral neuropathy tends to affect sensory nerve function more severely, particularly in the lower extremities. Patients excluded from the study due to absent waveforms exhibited a higher rate of absent waveforms in the sural nerve, indicating extensive involvement of lower extremity nerve fibers in neuropathic lesions. Several studies support the notion that sensory nerve involvement is the most sensitive indicator of subclinical diabetic neuropathy. A recent study reached a similar conclusion, stating that sensory nerves are more susceptible to metabolic alterations in the pathogenesis of diabetic neuropathy. This vulnerability is attributed to the fact that sensory nerves are thinner and longer compared to motor nerves [21]. It is essential to acknowledge that there have been some studies contradicting our findings and suggesting that changes in motor nerves were more frequent than changes in sensory nerves in the context of diabetic neuropathy [22,23]. These studies might be explained by the significantly longer duration of diabetes in their patient population, resulting in severe and advanced sensory-motor neuropathy. Longer duration of diabetes results in abnormal electrophysiology disturbances, as reported in a study conducted on 700 patients with long-duration diabetic peripheral neuropathy [24]. Another study conducted on 50 diabetic children with peripheral neuropathy in Sudan reported motor involvement in 68.2% of the children and sensorimotor involvement in 31.8%, while none had pure sensory involvement [25]. It is worth noting that their study focused on type 1 diabetic children and had a smaller sample size compared to our study.

The electrophysiological abnormalities observed in the present study demonstrate that the most common nerve derangement observed was the sensory nerve action potential of the sural nerve, followed by the sensory nerve action potential of the median nerves. Sensitivity analysis in our study also revealed the dominance of reduced sensory nerve action potential over reduced conduction velocity. The congruence between our findings and the study by Bi et al., 2008, which emphasized the sensitivity of sensory nerve action potential amplitude in diagnosing mild or early diabetic peripheral neuropathy, further supports the importance of considering sensory nerve parameters in the assessment of neuropathy [26]. Another study by Weisman and colleagues showed a similar result and stated that the best determinants of neuropathy were threshold values for sural amplitude potential and peroneal conduction velocity [27].

The observed findings strongly suggest that axonal degeneration may be the predominant histological abnormality in early diabetic neuropathy. This theory aligns with the potential mechanisms underlying axonal degeneration, which can be attributed to various metabolic derangements induced by diabetes itself. These metabolic disturbances, including oxidative stress, advanced glycation end-products (AGE) mediated injury, and aldose reductase pathways, can collectively lead to impairments in axoplasmic transport within nerves. As a consequence, distal axons may not receive sufficient nutrition, eventually leading to their degeneration. This proposed mechanism is in line with previous research, such as a study conducted in 40 diabetic children with an average diabetes duration of 6.63 ± 0.25 years, which also identified axonal degeneration as the most common cause of neuropathy [28]. The convergence of findings from different studies provides further support to the hypothesis that axonal degeneration plays a significant role in the pathogenesis of diabetic neuropathy.

The evidence from the current study supports the routine use of nerve conduction studies for the early diagnosis of diabetic peripheral neuropathy. Along with standard nerve conduction study indices, the amplitude of sensory nerve action potential should be routinely analyzed in diabetic patients, as it has been found to be the most sensitive index of neuropathy, even in asymptomatic diabetic neuropathy [20,29,30]. This approach will help in the early detection and management of diabetic peripheral neuropathy and prevent long-term complications like limb amputation.

This study has a few limitations. Firstly, its cross-sectional design restricts our ability to examine the time course of the observed differences and establish a causal relationship. It is important to consider longitudinal studies to gain a deeper understanding of the development and progression of the identified abnormalities. Additionally, a limitation of this study is the lack of pathological evidence to confirm axonal degeneration as the underlying cause of the decreased amplitude of sensory and motor action potentials. Obtaining such evidence would require invasive procedures, such as sural nerve biopsy, which are not well tolerated by patients. Therefore, while the observed changes in nerve conduction parameters strongly suggest axonal degeneration, future studies with histopathological confirmation would provide a more comprehensive understanding of the underlying pathology. Despite these limitations, the findings of this study contribute valuable insights into the early detection and characterization of diabetic peripheral neuropathy.

## Conclusions

The current study indicates that varying degrees of peripheral neuropathy are present in both sensory and motor nerves during the early stages of diabetes, even before the appearance of noticeable symptoms. The findings contribute valuable insights into the pathophysiology of diabetic peripheral neuropathy, emphasizing the importance of early detection and intervention to prevent or mitigate its progression. By prioritizing routine nerve conduction assessments in diabetic patients, we can effectively identify neuropathic changes and implement timely treatments, ultimately improving the quality of life for those affected by this condition. Continued research in this field, specifically exploring advancements in antioxidant therapies, will be instrumental in advancing our knowledge and refining clinical approaches to managing diabetic neuropathy.
